# The Application of mRNA Technology for Vaccine Production—Current State of Knowledge

**DOI:** 10.3390/vaccines13040389

**Published:** 2025-04-04

**Authors:** Anna Paczkowska, Karolina Hoffmann, Agata Andrzejczak, Weronika Faustyna Pucek, Dorota Kopciuch, Wiesław Bryl, Elżbieta Nowakowska, Krzysztof Kus

**Affiliations:** 1 Department of Pharmacoeconomics and Social Pharmacy, Poznan University of Medical Sciences, Rokietnicka 3 Street, 60-806 Poznan, Poland; a-andrzejczak00@wp.pl (A.A.); weronikapucek@o2.pl (W.F.P.); dkoligat@ump.edu.pl (D.K.); kkus@ump.edu.pl (K.K.); 2 Department and Clinic of Internal Diseases and Metabolic Disorders, Poznan University of Medical Sciences, Przybyszewskiego 49 Street, 60-355 Poznan, Poland; karolinahoffmann@ump.edu.pl (K.H.); wieslawbryl@wp.pl (W.B.); 3 Department of Pharmacology and Toxicology Institute of Health Sciences, Collegium Medicum, University of Zielona Gora, Licealna 9 Street, 65-417 Zielona Góra, Poland; elapharm@ump.edu.pl

**Keywords:** mRNA technology, vaccines, immunotherapy

## Abstract

Over the past 20 years, intensive research has been conducted on the development of therapeutic mRNA, leading to numerous discoveries that have enabled its use in therapy. The main achievements in this field include increasing mRNA stability, reducing its immunogenicity (i.e., its ability to trigger an immune response), and solving the challenge of delivering mRNA into cells—all to achieve a therapeutic effect. The aim of this study was to review the scientific literature on the use of mRNA technology in the production of vaccines. Various methods of applying mRNA technology that could potentially be introduced into clinical practice in the future are described. A detailed analysis was conducted on the approved COVID-19 vaccines developed by Pfizer/BioNTech (New York, NY, USA) and Moderna (Kirkland, QC, Canada), as their introduction marked a groundbreaking moment in the advancement of mRNA technology. This study was based on the latest scientific literature from reputable publishers and medical databases such as PubMed and ClinicalTrials. In conclusion, mRNA technology is currently experiencing rapid development, significantly driven by the ongoing COVID-19 pandemic. The application of this technology holds great potential not only for vaccines against infectious diseases but also for cancer treatment. However, further research is necessary to facilitate its broader clinical implementation.

## 1. Introduction

Messenger RNA (mRNA) is a relatively small single-stranded structure with a length of approximately 1000–5000 nucleotides. A single mRNA molecule typically consists of five essential elements necessary for its expression: a cap structure (CAP), a 5′ untranslated region (5′ UTR), an open reading frame (ORF) encoding the gene of interest, flanked by a START and STOP codon, a 3′ untranslated region (3′ UTR), and a poly(A) tail at the 3′ end, which consists of approximately 100–250 adenosine residues [1].

RNA polymerase is an enzyme that enables the transcription of mRNA from DNA in living cells. Subsequently, mRNA undergoes splicing and is transported from the nucleus to the cytosol, where ribosomes facilitate the expression of encoded proteins. Ribonucleic acid is significantly less stable than deoxyribonucleic acid. This is partly due to the presence of a 2′OH (hydroxyl) group in ribose, which makes ribose hydrolytically less stable than deoxyribose. RNA is also much more susceptible to oxidation and enzymatic degradation [2].

Synthetic mRNA, on the other hand, is produced through in vitro transcription (IVT) from linearized plasmid DNA (pDNA) or a PCR template containing the gene of interest and a promoter region for the RNA polymerase of bacteriophage T7, SP6, or T3. This reaction requires four ribonucleotide triphosphates (NTPs), an RNase inhibitor (to inactivate RNases), a buffer with an appropriate pH, and magnesium ions as cofactors for RNA polymerase. After the IVT reaction is completed, the mRNA product must be purified from residual reaction components (unused NTPs, RNA polymerase). The prepared mRNA is then ready for further use [1].

Issues related to mRNA stability, immunogenicity, and intracellular delivery challenges led to the late introduction of mRNA-based therapies. In recent years, extensive research has been conducted not only on the structure of the molecule itself but also on improving suitable carriers to overcome these obstacles. By modifying the mRNA backbone structure, such as optimizing the 5′ and 3′ regions or adding a poly(A) tail, the stability of the molecule can be significantly enhanced. Additionally, selecting an appropriate viral or non-viral carrier (with non-viral carriers proving to be much safer) is crucial for the safety of the formulation and its fate within the body. So far, lipid nanoparticles have been identified as the most effective method for delivering mRNA into cells [3].

The mRNA molecule was discovered in 1961 by two French scientists, Jacques Monod and François Jacob. However, nearly 30 years later, in 1990, research on the use of conventional mRNA in therapy began. This was a preclinical study involving the direct injection of a gene into mouse muscle tissue in vivo [4].

The use of self-amplifying mRNA (saRNA) was first reported in 1994 when it was discovered to be a promising candidate for the development of various types of vaccines [1]. Self-amplifying mRNA is characterized by increased and prolonged protein expression. A significant difference between conventional mRNA and saRNA is their size. Self-amplifying mRNA is a much larger and more complex molecule, consisting of approximately 9000–12,000 nucleotides. In addition to the fundamental elements present in mRNA, saRNA also contains sequences encoding a viral replicase complex, as well as genomic and subgenomic promoters.

Most saRNA molecules are based on the genome of alphaviruses, which are small, enveloped, positive-sense RNA viruses that produce large amounts of subgenomic RNA encoding viral structural proteins during their natural replication cycle in the cytosol of host cells. The substantial size of saRNA contributes to challenges in its cellular delivery. Moreover, beyond encoding the protein of interest, saRNA also encodes viral proteins that may be potentially immunogenic [1].

mRNA technology, utilizing both conventional mRNA and saRNA, has significantly advanced due to the ongoing SARS-CoV-2 pandemic. It is being explored not only for vaccines against infectious diseases and cancer but also for protein replacement therapies, gene editing, and many other applications.

This paper presents examples of the application of this technology in vaccine and drug production. The aim of this work was to review the literature on the use of mRNA technology in vaccine development.

### Current State of Research on mRNA Delivery Systems

Delivering therapeutic messenger RNA (mRNA) into the human body has become a pivotal area of research, especially with the success of mRNA-based vaccines. Several delivery methods have been developed to ensure the stability and efficacy of mRNA therapies:

Lipid Nanoparticles (LNPs): LNPs are the most established mRNA delivery vehicles, encapsulating mRNA to protect it from degradation and facilitating cellular uptake. They have been instrumental in the development of mRNA vaccines, such as those for COVID-19. Recent advancements include optimizing LNP formulations to enhance delivery efficiency and reduce side effects. For instance, studies have focused on tailoring LNP compositions to improve targeted delivery and minimize immunogenicity [5].

Polymeric Nanoparticles: These involve polymers like chitosan or poly(lactic-co-glycolic acid) (PLGA) to form nanoparticles that can deliver mRNA. Research has demonstrated that chitosan-coated PLGA nanoparticles can effectively deliver mRNA to the lungs, suggesting potential for treating respiratory diseases [6].

Extracellular Vesicles (EVs): EVs are naturally occurring particles that transport biomolecules between cells. Scientists have engineered EVs to carry mRNA, leveraging their innate biocompatibility and ability to evade immune detection. A study demonstrated that EV-encapsulated mRNA could initiate collagen production in photoaged skin, highlighting their therapeutic potential [7].

Gene Editing Tools: mRNA can be used to encode programmable nucleases, such as CRISPR-Cas9, facilitating targeted genome modifications. This approach enables correction of genetic mutations or introduction of protective changes, offering potential treatments for various genetic disorders [6].

Each of these delivery methods is under active investigation to enhance their safety, targeting specificity, and overall therapeutic efficacy.

## 2. mRNA-Based Vaccines

The outbreak of the SARS-CoV-2 pandemic significantly contributed to the advancement of research on the use of mRNA technology in vaccines. Compared to other types of vaccines, such as protein subunit vaccines, viral vector vaccines, or DNA-based vaccines, mRNA vaccines offer several advantages, including improved safety. Messenger RNA does not integrate into the host cell genome; instead, it serves only as a temporary carrier of information necessary for protein synthesis.

One of the challenges in using naked mRNA is its difficulty in crossing lipid membranes due to its large molecular size, hydrophilicity, negative charge, and fragility. For this reason, mRNA in vaccines is often enclosed within a carrier (e.g., lipid nanoparticles, which are among the most effective options) before being administered into the body [3].

One of the advantages of mRNA-based vaccines is that they can be produced in a cell-free manner, enabling rapid and cost-effective manufacturing. Additionally, a single mRNA vaccine can encode multiple antigens, enhancing the immune response against various pathogens. This feature is particularly important during the ongoing SARS-CoV-2 pandemic, as it allows vaccines to provide protection against newly emerging virus mutations [8].

Human coronaviruses were first discovered in the 1960s, and their name originates from the spike protein structure on their surface. The severe acute respiratory syndrome coronavirus (SARS-CoV) belongs to the Coronaviridae family within the order Nidovirales. These viruses primarily target the respiratory system but can also affect the intestines, liver, and nervous system [9].

Effective and safe COVID-19 vaccines should generate both humoral and cellular immunity against SARS-CoV-2 while minimizing adverse side effects. Like any other drug, a vaccine must undergo a series of preclinical and clinical trials before being approved for use [10].

### SARS-CoV-2 mRNA Vaccines

So far, only two mRNA-based vaccines have been authorized: those developed by Moderna and Pfizer/BioNTech. The first vaccine to be introduced was Pfizer/BioNTech’s, which received approval by FDA on (11 December 2020). Nearly ten days later, on 21 December, it was authorized for use in the European Union. This vaccine, marketed under the name COMIRNATY (clinical name BNT162b2), contains nucleoside-modified mRNA encoding the spike protein, enclosed in lipid nanoparticles. It is administered in two doses, with the second dose given at least 21 days after the first. A person is considered fully vaccinated only after receiving both doses [11].

According to the European Commission’s guidelines, every approved vaccine is subject to pharmacovigilance (safety monitoring). This includes submitting a Periodic Safety Update Report (PSUR) to the European Medicines Agency (EMA) on a monthly basis, particularly during the first six months after market introduction [12].

Recent studies conducted by Pfizer/BioNTech indicate that a booster dose significantly enhances vaccine effectiveness—by approximately 95%. In this study, participants had been fully vaccinated with Comirnaty for at least 11 months before receiving the booster. Based on these findings, it is recommended that individuals receive a booster dose no later than one year after their initial vaccination [12].

On 6 January 2021, the EMA issued a positive opinion for the conditional marketing authorization of Moderna’s vaccine (approval by FDA on 18 December 2020), Moderna COVID-19 Vaccine (clinical name: mRNA-1273), for use in the EU. This vaccine also contains nucleoside-modified mRNA encoding the spike protein, enclosed in lipid nanoparticles. Full vaccination requires two doses, with the second dose administered 28 days after the first. Both vaccines have demonstrated high efficacy in preventing COVID-19 infection and have shown a favorable safety profile. However, storage conditions pose challenges: these vaccines must be kept in specialized laboratory freezers at extremely low temperatures. BNT162b2 (Comirnaty) must be stored at −60 °C for up to 6 months; 2–8 °C for up to 5 days, while mRNA-1273 (Moderna) can be stored at −15 °C or lower for up to 6 months; 4–8 °C for up to a month and at room temperature for up to 12 h [13,14].

## 3. Mechanism of Action of mRNA and Vector-Based COVID-19 Vaccines

Traditional vaccines work by delivering ready-made antigens to the body in the form of attenuated (weakened) microorganisms (“live” vaccines), inactivated (killed) pathogens, or isolated proteins (subunit vaccines). In contrast, vaccines containing messenger RNA (mRNA) or genetically modified viral vectors deliver genetic material into the cells of the vaccinated person. This genetic material encodes a specific antigen of the pathogen (the “vaccine antigen”), which the host’s own cells then produce. Thus, instead of containing the actual antigen or pathogen, these vaccines provide precise “instructions” for its production [15].

In cellular biology, mRNA is an intermediate molecule that transfers genetic information from DNA in the nucleus to the cytoplasm, where proteins are synthesized. Vaccine mRNA, however, is produced in vitro through a transcription reaction. Genetic information is transcribed from linear DNA to RNA using an enzyme called RNA polymerase. The resulting RNA undergoes enzymatic modifications to ensure it resembles mature mRNA molecules naturally found in human cells. These modifications allow the vaccine mRNA to serve as a proper template for protein synthesis. Importantly, vaccine mRNA cannot re-enter the cell nucleus from the cytoplasm.

To protect mRNA from rapid degradation after injection, it is encapsulated in delivery systems such as lipid nanoparticles (LNPs), which facilitate cellular uptake via endocytosis [16]. These particles primarily enter muscle cells and antigen-presenting cells (APCs), such as dendritic cells, near the injection site. Once inside the cytoplasm, the mRNA is used as a template for protein biosynthesis. The produced proteins undergo post-translational modifications and are transported based on their biochemical properties. Shortly after translation, the mRNA molecule is degraded by naturally occurring cytoplasmic ribonucleases. After mRNA vaccination, the host’s cells produce the vaccine antigens in the same way they would during a natural infection. These antigens are then presented as short peptides on MHC class I or II molecules, leading to stimulation of both humoral and cellular immune responses. As a result, mRNA and vector-based vaccines generate a level of immunogenicity and efficacy similar to those of live attenuated vaccines, but with a better safety profile [17].

## 4. Safety Advantages of mRNA and Vector-Based Vaccines

Unlike live vaccines, which are generally not recommended for immunocompromised patients or pregnant women due to a slight risk of reversion to a virulent form, mRNA and vector vaccines are considered safer. The Comirnaty (Pfizer/BioNTech) vaccine contains mRNA produced via in vitro transcription, encoding the full-length SARS-CoV-2 spike (S) protein. To enhance the protein’s stability, two proline residues were introduced into the sequence, ensuring that the S protein remains in its prefusion conformation—its natural state before binding to host cell receptors. Additionally, the vaccine’s mRNA sequence has been optimized for codon usage and modified by incorporating 1-methylpseudouridine, an analog of uridine [18].

Messenger RNA (mRNA) and vector-based vaccines have emerged as pivotal tools in modern immunization strategies, each offering distinct safety advantages.

mRNA Vaccines:

1. Non-Integrative Nature: mRNA vaccines function by delivering a transient blueprint for antigen production without integrating into the host genome, thereby eliminating risks associated with insertional mutagenesis.
2. Rapid Degradation: The inherent instability of mRNA ensures its swift degradation within the body, reducing concerns about long-term persistence and associated adverse effects.
3. No Risk of Infection: Unlike traditional vaccines that may use attenuated pathogens, mRNA vaccines are non-infectious, thereby eliminating the possibility of vaccine-induced disease.
4. Controlled Manufacturing: The cell-free synthesis of mRNA vaccines minimizes contamination risks and allows for precise control over production processes, enhancing overall safety [19].

Vector-Based Vaccines:

1. High Immunogenicity: Viral vectors, such as adenoviruses, are adept at inducing robust immune responses without the need for adjuvants, enhancing vaccine efficacy.
2. Non-Replication in Humans: Many viral vectors are engineered to be replication-deficient in human cells, ensuring they cannot cause disease
3. Established Safety Profiles: Vectors like vaccinia virus have a long history of safe use in vaccines, providing a foundation of trust and understanding in their application [20].

Studies conducted by Paczkowska et al. [21] on the comparative assessment of the safety (prevalence of both local and systemic side effects) of COVID-19 vaccines (Pfizer–BioNTech, Moderna, Oxford–AstraZeneca) among healthcare workers (doctors, nurses, and pharmacists) have shown that the short-term safety profiles of the eligible COVID-19 vaccines (Pfizer–BioNTech, Moderna, Oxford–AstraZeneca) are acceptable. the occurrence of side effects following a SARS-CoV-2 vaccine administration was reported by 53.11% of respondents vaccinated with BNT162b2 (Pfizer–BioNTech), 72% of those vaccinated with mRNA-1273 (Moderna), and 67.59% of those vaccinated with ChAdOx1-S (Oxford–AstraZeneca). The most common local or systemic side effects regardless of the type of vaccine received were pain at the injection site (49.93%—BNT162b2, 69.33%—mRNA-1273, 53.10%—ChAdOx1-S), headache (28.89%—BNT162b2, 50.00%—mRNA-1273, 42.76%—ChAdOx1-S), muscle pain (25.00%—BNT162b2, 43.33%—mRNA-1273, 46.90%—ChAdOx1-S), fever (16.57%—BNT162b2, 42.67%—mRNA-1273, 51.72%—ChAdOx1-S), chills (19.67%—BNT162b2, 39.33%—mRNA-1273, 46.21%—ChAdOx1-S) and fatigue (30.18%—BNT162b2, 39.33%—mRNA-1273, 33.10%—ChAdOx1-S). The number and intensity of reported side effects following administration of a BNT162b2 (Pfizer–BioNTech) vaccine were significantly lower than in the other two study groups (*p* < 0.00001).

Risk factors for side effects following administration of one of the SARS-CoV-2 vaccines subject to the analysis included being female, young, and suffering from a diagnosed allergy. Similar to other medications, allergic reactions can occur during vaccination. While most reactions are neither frequent nor serious, anaphylactic reactions are potentially life-threatening allergic reactions that are encountered rarely but can cause serious complications. Reactions are more often caused by inert substances, called excipients, which are added to vaccines to improve stability and absorption, increase solubility, influence palatability, or create a distinctive appearance, and not by the active vaccine itself. The excipients mostly incriminated for allergic reactions are polyethylene glycol, also known as macrogol, found in the currently available Pfizer–BioNTech and Moderna COVID-19 mRNA vaccines, and polysorbate 80, also known as Tween 80, present in Oxford–AstraZeneca and Johnson & Johnson COVID-19 vaccines [15]. Therefore, people suffering from allergies have a greater risk of side effects following a COVID-19 vaccination. On the basis of data from the Vaccine Adverse Event Reporting System (VAERS) and the V-safe system of the U.S. Centers for Disease Control and Prevention (CDC), the rates of non-serious AEFI after public administration of BNT162b2 and mRNA-1273 were similar to the clinical trials [22]. Anaphylaxis occurs at a rate of approximately 1 case per million doses for the majority of vaccines, whereas the rates of anaphylaxis associated with BNT162b2 and mRNA1273 appear to be 4.7 times and 2.5 times higher, respectively [23,24]. Thrombosis and thrombocytopenia as a side effect of adenoviral vector vaccines (ChAdOx1 nCoV-19 and Ad26.COV2.S) were noted, including several deaths and severe outcomes [25,26,27,28].

Yasuhara et al. [29] conducted a systematic review and meta-analysis to examine the frequency, clinical features, and early outcomes associated with myopericarditis after COVID-19 mRNA vaccination in adolescents and young adults.

In this systematic review and meta-analysis of 23 studies, including 854 patients aged 12 to 20 years with vaccine-associated myopericarditis, the incidence of myopericarditis was higher in males after the second dose. Although 15.6% of patients had left ventricular (LV) systolic dysfunction, only 1.3% had severe LV systolic dysfunction (ejection fraction <35%); late gadolinium enhancement was found in 87.2%, and 23.2% required intensive care unit admission; however, no in-hospital mortality was observed.

These findings suggest largely favorable outcomes of COVID-19 vaccine-associated myopericarditis in adolescents and young adults.

## 5. Role of 1-Methylpseudouridine in Vaccine mRNA

1-Methylpseudouridine is a naturally occurring nucleoside found in cellular mRNA. Its inclusion in the vaccine’s mRNA improves protein translation efficiency in vivo while reducing inflammatory responses triggered by the body’s Toll-like receptors (TLRs) detecting foreign RNA. The use of modified nucleotides thus enhances vaccine safety by minimizing excessive innate immune activation [30].

## 6. Efficient and Rapid mRNA Vaccine Production

mRNA vaccines stimulate cellular immune responses, leading to the elimination of antigen-presenting cells via cytotoxic lymphocytes near the injection site. The vaccine mRNA is produced in vitro using biochemical synthesis, where genetic information is transcribed from linear DNA using RNA polymerase. The resulting mRNA undergoes enzymatic modifications to include a 5′ guanine cap and a 3′ poly-A tail, mimicking mature cellular mRNA. The final product is purified and encapsulated in lipid nanoparticles (LNPs). mRNA vaccine production does not involve cell cultures, making the manufacturing process fast and highly efficient [30].

## 7. Influenza Vaccines

Influenza viruses cause some of the most virulent respiratory infections in humans. They are responsible for approximately 290,000 to 650,000 deaths annually worldwide [31]. Traditional flu vaccines target the hemagglutinin protein of the virus. The virus mutates rapidly, requiring annual reviews and modifications of the hemagglutinin antigen component in vaccines. Currently, the vaccines used are inactivated flu viruses grown in chicken eggs. These vaccines are characterized by long production times, purification difficulties, and mutations that contribute to reduced effectiveness. Scientists have begun researching alternative vaccine production methods. It is hypothesized that a vaccine based on mRNA technology would not require annual modifications, as it would provide immunity against multiple strains and subtypes of the flu [8]. Research is currently underway on vaccines utilizing both self-amplifying mRNA and conventional mRNA.

Traditional influenza vaccines are formulated annually to protect against up to four circulating strains of the virus. However, predicting which strains will dominate each season is challenging, often leading to mismatches and reduced vaccine efficacy. mRNA vaccine platforms offer a significant advantage in this context. They can incorporate mRNA sequences encoding hemagglutinin (HA) antigens from all known influenza subtypes, enabling the production of a single vaccine that provides broad protection. This approach has been demonstrated in preclinical studies, where an experimental mRNA vaccine elicited strong antibody responses against all 20 known influenza A and B virus subtypes in animal models [32].

Unlike traditional inactivated virus vaccines, which present the entire viral proteome—including non-neutralizing or highly variable regions—mRNA vaccines can be designed to express only specific viral antigens that are conserved and elicit neutralizing antibodies. This targeted approach focuses the immune response on critical components of the virus, potentially enhancing vaccine efficacy and breadth of protection. For instance, by selecting conserved HA antigens, mRNA vaccines can induce immunity that is effective across diverse influenza strains [33].

The self-amplifying RNA (saRNA) vaccine can be delivered as plasmid DNA, virus-like RNA particles, or in vitro transcribed RNA. SaRNA-based vaccines have induced strong immune responses in preclinical studies. These vaccines are derived from the genome of alphaviruses, in which the genes encoding the RNA replication mechanism are intact. Studies conducted on mice have shown that immunization with 10 mg of saRNA vaccine encoding PR8 H1N1 IAV HA induced an antibody response and protection against lethal infection. PR8 H1N1 IAV HA refers to the hemagglutinin (HA) protein of the H1N1 subtype of the influenza A virus (IAV), specifically derived from the A/Puerto Rico/8/1934 (PR8) strain. The HA protein is a surface glycoprotein that plays a key role in virus entry into host cells by mediating binding to sialic acid receptors and subsequent membrane fusion. Furthermore, the HA protein is a primary target for neutralizing antibodies, making it a focal point of interest in vaccine design and immunological studies [34]. In turn, the PR8 strain has been widely used in vaccine development due to its well-characterized genetics and stable growth in laboratory settings [35]. The mRNA delivery method proved crucial for achieving the best immune response. The most effective delivery method was lipid nanoparticles, which coated the mRNA and protected mice from lethal viral infection when administered intramuscularly [31].

Conventional mRNA vaccines can be produced using various modified nucleosides, which are important in increasing stability, reducing innate immune activation, and increasing protein translation. Studies are ongoing on the administration of conventional mRNA encapsulated in lipid nanoparticles against the influenza virus. Lee at al. reported that intradermal immunization of mice, ferrets, and pigs with different mRNA vaccines encoding IAV HA, NP (nucleoprotein), and NA (neuraminidase) proteins induced a protective immune response after a single vaccination [31]. However, these studies primarily utilized non-modified nucleosides. Lee et al. [31] also showed that intravenous immunization of mice with a vaccine encoding PR8 H1N1 IAV HA also led to increased cell activation after a single dose, though it is unclear whether modified nucleosides were used. The key question is how these findings translate to clinical trials [8]. The first directly injectable mRNA vaccine against H10N8 and H7N9 subtypes was developed by Moderna. The vaccines were administered in a phase 1 trial with two intramuscular injections containing mRNA with modified nucleosides, encapsulated in lipid nanoparticles [36]. The inclusion of modified nucleosides likely contributed to the observed safety and tolerability, minimizing adverse effects like headaches, fatigue, and chills [8,36].

For the first RNA flu vaccine trial against seasonal human influenza, CureVac developed a strategy using mRNA in lipid nanoparticles with a modified sequence, which enabled a high level of protein translation in vivo without the use of modified nucleosides. This study demonstrated that a single intramuscular vaccination induced immunity in primate mammals. Furthermore, a second dose effectively boosted the immune response [31].

Preclinical and clinical studies have shown that mRNA technology holds great potential and may prove to be the best option for acquiring immunity against influenza viruses. Three phases of clinical trials for the first dual mRNA vaccine against influenza and COVID-19 have been successfully completed and shown to be as effective as separate vaccines against these infections. Clinical trials involving 8000 volunteers, at least 50 years old (with half of the participants over 64 years old), indicated that Moderna’s dual vaccine offers the same or even greater immunity against both influenza and COVID-19. This age group was chosen because people over 50 are more susceptible to complications from both diseases. Research is also underway with younger individuals, as the vaccine will be targeted to them as well. The side effects of the dual mRNA vaccine are generally mild. There may be a slight reaction at the injection site, and vaccinated individuals might experience mild fatigue [37].

### Avian Influenza Vaccines

Avian influenza, particularly the highly pathogenic H5N1 strain, continues to pose a significant global threat.

In 2024, the United States confirmed the first case of human infection with avian influenza virus transmitted from an infected dairy cow. This marked the first observed case of the virus spreading to a human from another mammal. However, the infection in this individual was mild. The U.S. Centers for Disease Control and Prevention (CDC) reported that avian influenza generally has a mild course in humans, typically causing upper respiratory tract symptoms or conjunctivitis, with pneumonia being less common. However, it remains uncertain whether the avian influenza virus will mutate, potentially leading to more severe symptoms in infected individuals [38].

Traditional inactivated vaccines have limitations, including the need for egg-based production and slower adaptability to emerging strains. The mRNA vaccine platform offers a promising alternative due to its rapid production, high adaptability, and ability to elicit strong immune responses.

Recent studies have demonstrated the feasibility of mRNA vaccines against avian influenza. Arevalo et al. [32] reported the development of a multivalent nucleoside-modified mRNA vaccine capable of inducing broad protection against multiple influenza virus subtypes, including H5N1.

According to the latest media reports, Moderna has been conducting intensive research since 2023 to develop a vaccine against avian influenza caused by the H5N1 virus. This initiative was prompted by the increasing number of avian influenza cases among birds and livestock. There is currently no indication that the virus is being transmitted between humans. According to Moderna, developing a vaccine provides protection against a potential pandemic [39].

Additionally, the World Health Organization (WHO) has announced the launch of a new project aimed at accelerating the development and accessibility of an mRNA-based avian influenza (H5N1) vaccine for manufacturers in low- and middle-income countries. The project is led by the Argentine manufacturer Sinergium Biotech [38].

## 8. HIV Vaccines

Human Immunodeficiency Virus (HIV) belongs to the Retroviridae family, which integrates its genome into the host DNA through a series of complex stages. HIV can be divided into two types—HIV-1, the globally prevalent type, and HIV-2, which is dominant in Eastern and Central Africa but also appears in Europe and India. Both types of the virus can lead to the development of AIDS (Acquired Immunodeficiency Syndrome) [40]. The ongoing COVID-19 pandemic has reminded researchers of the importance of further work to understand the mechanisms of viruses, particularly HIV. The disease caused by HIV—AIDS—was first described over 40 years ago, and despite significant advances, no effective therapy or vaccine has been developed to prevent infection [41].

The primary routes of HIV transmission are sexual contact, contact with infected blood, and vertical transmission, which occurs during pregnancy, childbirth, or breastfeeding from an infected mother. The World Health Organization (WHO) reported that 1.5 million people were infected with HIV last year, and over 700,000 people died from AIDS-related complications [42]. An ideal HIV vaccine should elicit both humoral and cellular immunity. Neutralizing antibodies form the first layer of protection, preventing the infection of host cells when the virus enters the body. If some virions manage to evade neutralizing antibodies, the secondary defense layer would consist of cytotoxic CD8+ T lymphocytes, which eliminate the earliest infected cells, preventing the formation of a latent reservoir of infected cells [43].

Most HIV vaccine research has focused on protein subunits, and significant efforts have also been made with DNA-based vaccines, although they have not yet demonstrated real-world efficacy [44]. While the success of mRNA vaccines against COVID-19 has highlighted the potential of mRNA technology, it is important to recognize that HIV poses unique challenges, including latency, rapid mutation, and direct targeting of the immune system [45]. These factors have historically hampered vaccine development efforts.

However, mRNA-based HIV vaccines are being investigated for their ability to induce broadly neutralizing antibodies (bnAbs) and stimulate robust cellular immunity, potentially offering advantages over previous strategies [16]. Although it is still uncertain whether mRNA vaccines can bypass HIV immune evasion mechanisms, preliminary studies have shown promising immunogenicity and the ability to generate desirable immune responses in preclinical and early clinical phase studies [46]. For example, a multiclade mRNA env–gag VLP vaccine has been shown to induce HIV-1 level 2 neutralizing antibodies and reduced the risk of heterologous SHIV infection in macaques [46]. Moreover, an ongoing collaboration between IAVI and Moderna aims to investigate mRNA-based HIV vaccine candidates, reflecting ongoing research efforts to overcome these challenges [47]. It should be underlined that further research is needed to determine the full potential of mRNA technology in overcoming the complex challenges of HIV vaccine development [48].

The table above (Table 1) summarizes various methods of delivering mRNA vaccines against HIV that have been researched in recent years. However, further detailed studies are required before any of these methods can advance to the next phase of clinical trials and potentially be approved for use in the future [40,41,42,49].

### Respiratory Syncytial Virus mRNA Vaccine

In May 2024, the U.S. Food and Drug Administration (FDA) approved Moderna’s mRNA-based vaccine, mResvia, for the prevention of respiratory syncytial virus (RSV) in adults aged 60 and older. This approval marked a significant advancement in RSV prevention, offering a new tool to protect older adults from this respiratory pathogen.

RSV is a highly contagious seasonal respiratory virus and a leading cause of lower respiratory tract infections and pneumonia. It causes a particularly high disease burden in infants and the elderly. Each year in the United States, up to 160,000 elderly individuals are hospitalized, and 10,000 die due to RSV infection [50].

The approval of the mRESVIA vaccine by the FDA was based on positive data from the Phase 3 clinical trial, ConquerRSV. This global study involved approximately 37,000 adults aged 60 years or older across 22 countries. The primary analysis, with a median follow-up of 3.7 months, showed the vaccine’s efficacy against RSV lower respiratory tract disease (LRTD) at 83.7% (95.88% CI 66.0%, 92.2%). These results were published in The New England Journal of Medicine [51].

During the FDA review, a supplementary analysis of the primary endpoint was conducted. It included cases that began before the cutoff date of the primary analysis but were not previously confirmed. The results were consistent with the primary analysis [VE 78.7% (CI 62.9%, 87.8%)] and were included in the package insert in the United States. An additional long-term analysis demonstrated continued protection against RSV LRTD over a median follow-up of 8.6 months [51].

No serious safety concerns were identified in the Phase 3 study. The most commonly reported adverse events were injection site pain, fatigue, headache, muscle aches, and joint pain [51].

The development and approval of mResvia underscore the potential of mRNA technology in addressing various infectious diseases beyond COVID-19, highlighting the adaptability and rapid development capabilities of mRNA-based vaccines.

## 9. Cancer Vaccines

The use of mRNA technology to create cancer vaccines has recently become an increasingly popular method for treating cancers. This is a promising alternative to currently available cancer therapies, as mRNA vaccines can be designed to target and kill cancer cells while sparing healthy ones [31]. Most cancer vaccines are used as treatments rather than for prevention. However, they have the disadvantage of not being effective against every type of cancer. Despite this, they offer great hope for a potential cure for patients [52].

The importance of mRNA technology in the creation of cancer vaccines lies in the fact that the so-called “antigen drift” that occurs in cancer cells limits the effectiveness of traditional vaccines [52]. Examples of preventive vaccines that help prevent cancer development include vaccines against Hepatitis B virus (HBV) and Human Papillomavirus (HPV) [52]. The introduction of these vaccines has had a significant impact on reducing cancer rates. In the case of the HBV vaccine, it has reduced the incidence of hepatocellular carcinoma in children [53], and in the case of the HPV vaccine, it has reduced the incidence of cervical cancer in women [54].

For therapeutic vaccines, personalized neoantigens are often used in their design [52]. The cancer-specific neoantigens are processed and presented on major histocompatibility complex (MHC) molecules and these mutated epitopes that are recognized by T cells are called “neoepitopes”. Neoepitopes are not expressed on the normal tissue and hence are recognized as foreign by the host’s immune system, and thus can elicit T cell immunity against tumors [52].

Neoantigen-based personalized vaccines can be in various formats including mRNA, peptide- and whole cell-based strategies. Kreiter et al. used three independent murine tumor models and show that the majority of the non-synonymous tumor mutations are recognized by CD4+ T cells and vaccination with such CD4+ immunogenic mutations induce potent anti-tumor responses [55]. The authors used NGS and bioinformatics and established a pipeline to select mutations as vaccine targets by prioritizing their expression levels and MHC class II-binding capabilities. Such a pipeline was used to produce synthetic poly-neo-epitope messenger RNA vaccines [55]. Vaccination of tumor-bearing mice with such polytope mRNA vaccines induced almost complete rejection of established tumors in vaccinated mice. Furthermore, this study showed that CD4+ T cell neo-epitope vaccination induced CD8+ CTL responses against an independent tumor antigen in the vaccinated mice, possibly due to the occurrence of an “antigenic spread” in the vaccinated mice [55].

Currently, many studies have been completed on mRNA vaccines for melanoma. Research conducted by the University Hospital in Belgium showed that a vaccine utilizing dendritic cells, which were electroporated with synthetic mRNA encoding the CD40 ligand, was safe and elicited the desired immune response [56]. The ligand of CD40, known as CD154 or CD40L, is a type II transmembrane protein, with a variable molecular weight between 32 and 39 kDa because of post-translation modifications. CD40L is expressed primarily by activated T cells, as well as activated B cells and platelets; and under inflammatory conditions is also induced on monocytic cells, natural killer cells, mast cells, and basophils. The wide expression of this costimulatory pair indicates the pivotal roles they play in different cellular immune processes [57].

Autologous monocyte-derived dendritic cells (DCs) electroporated with synthetic messenger RNA (mRNA) encoding a CD40 ligand, a constitutively active Toll-like receptor 4 and CD70, together with mRNA encoding fusion proteins of a human leukocyte antigen (HLA)-class II targeting signal (DC-LAMP) and a melanoma-associated antigen (MAA); either MAGE-A3, MAGE-C2, tyrosinase or gp100) (TriMixDC-MEL) are superiorly immunogenic. In conclusion study conducted by Wilgenhof et al. demonstrated that iv administration of TriMixDC-MEL is safe, feasible, immunogenic and results in encouraging durable clinical responses [56].

Other vaccines that have successfully completed phase I/II clinical trials include vaccines for cancers such as acute myeloid leukemia, prostate cancer, breast cancer, colorectal cancer, glioblastoma, and kidney cancer [31].

Researchers from the University of Antwerp in Belgium conducted a safety study on a vaccine containing dendritic cells electroporated with mRNA encoding the WT1 antigen (Wilm’s Tumor). The study showed that this vaccine is safe and can be administered intradermally to patients with acute myeloid leukemia [58]. Similarly, using dendritic cells as mRNA carriers for a specific antigen, researchers from the Netherlands studied a vaccine for colorectal cancer. The advantage of using dendritic cells is that they are professional antigen-presenting cells and part of the human immune system. This study aimed to check whether the injection of electroporated dendritic cells with CEA (carcinoembryonic antigen) mRNA antigen is a safe method and whether it outperforms the injection of dendritic cells presenting the CEA antigen. Patients with resectable liver metastases due to colorectal cancer were divided into two groups, depending on the vaccine administered, and were vaccinated three times a week. The results showed no significant difference in the effectiveness of both vaccines, suggesting that the CEA mRNA vaccine was not more effective [59].

CEA is a tumor-associated antigen that is expressed by almost all colorectal tumors. Hence, it is an attractive antigen to use in clinical immunization protocols. However, because CEA is also expressed in normal tissues, and it is also shed from the surface of tumor cells, a high threshold of tolerance must be overcome. Previously, it has been demonstrated that DCs loaded with CEA peptide can induce robust immune responses in colorectal cancer patients. Theoretically, using CEA mRNA instead of peptides to load the DCs may result in the induction of a much broader, more robust T-cell repertoire, since more epitopes are expressed and posttranslational modifications can occur. These results indicate that using CEA mRNA electroporation as a DC antigen loading strategy is not superior to peptide loading. The reasons for this difference may be twofold. Firstly, CEA is not processed through the MHC class II pathway whereas Gp100 is, unless it is coupled to a lysosomal targeting signal [60]. Secondly, a different T-cell repertoire may be present in the vaccinated patients. This study was not designed to allow meaningful conclusions on clinical efficacy. We did observe a transient increase in serum CEA upon vaccination in several patients, which may indicate a cytotoxic effect on CEA-expressing tumor cells [61].

The first clinical trial of a PSA mRNA—transfected DC cancer vaccine took place in 2002, highlighting the substantial effort needed to implement a new medical technology. The biggest breakthrough occurred in 2021 when the FDA approved the first mRNA vaccine (COVID-19 vaccine) [62]. As a result, BioNTech received approval from the FDA for the accelerated approval of a melanoma vaccine (clinical name BNT111) [63]. If all studies confirm the safety and efficacy of this vaccine, it could mark a significant breakthrough in treating advanced melanoma, offering hope to critically ill patients.

Moreover, recent phase I clinical trial data have demonstrated that mRNA neoantigen vaccination can lower the risk of pancreatic cancer recurrence [64]. The individualized mRNA vaccine autogene cevumeran was administered to patients with resected pancreatic ductal adenocarcinoma (PDAC). The vaccine induced tumor-specific T-cell responses that persisted for up to three years. It should be underlined, that study participants who exhibited vaccine-induced immune responses experienced delayed tumor recurrence compared to those without such responses. These findings highlight the potential of mRNA-based personalized immunotherapies in improving outcomes for PDAC patients [65].

Cancer vaccines are a crucial topic that needs to be explored further, as according to the Central Statistical Office’s data for 2024, cancer was one of the most frequent causes of death among Poles [66].

## 10. Conclusions

Based on the conducted literature review, it can be concluded that:

• mRNA technology has experienced rapid development in recent years.
• The use of mRNA technology in the production of vaccines against SARS-CoV-2 has significantly contributed to combating the ongoing COVID-19 pandemic.
• The first mRNA-based vaccine approved for use by the FDA and EMA was the Pfizer/BioNTech vaccine, Comirnaty.
• mRNA-based vaccines are strong candidates to replace traditional flu vaccines.
• mRNA technology may offer benefits for developing a vaccine against HIV.
• mRNA-based vaccine has demonstrated efficacy against melanoma.

Table 2 presents the application of mRNA technology for vaccine production in infectious diseases and cancer.

## Figures and Tables

**Table 1:** mRNA vaccine delivery methods Against HIV [40,41,42,49].

Delivery Technology	mRNA Type	In Vitro Test	In Vivo Test	Characteristics
Electroporation	saRNA, no nucleoside modifications	Dendritic cells (DCs)	Mouse	Specific antibodies and T-cell response higher than naked saRNA
Cationic micelles	Non-replicating RNA, no nucleoside modifications	Bone marrow-derived dendritic cells	Mouse	Induces maturation of bone marrow-derived dendritic cells, as well as T-cell response and specific IgG antibody production.
Cationic nanoemulsion	saRNA, no nucleoside modifications	Not conducted	Rabbit, Macaque	Induces IgG and neutralizing antibody titers even from small immunization doses
Polylactic acid (PLA) nanoparticles with cell-penetrating peptide (CPP)	Non-replicating RNA, no nucleoside modifications	Monocyte-derived dendritic cells	Not conducted	Induces maturation of monocyte-derived dendritic cells and secretion of pro-inflammatory cytokines
Lipid nanoparticles (LNP)	Non-replicating RNA, with nucleoside modifications	Not conducted	Rabbit, Macaque	Specific CD4+ and CD8+ T-cell response
Ex vivo delivery to dendritic cells (DCs)	Various	Monocyte-derived dendritic cells	Mouse	Induces maturation of monocyte-derived dendritic cells and cytotoxic T cells

**Table 2:** Summary the use of mRNA technology in vaccine development [67,68,69].

Study ID Numbers	Target Disease	Status	Manufacturer	Route of Administration
Infectious Diseases				
Comirnaty (BNT162b2)	SARS-CoV-2	Approved	Pfizer/BioNTech	I.M.
Spikevax (mRNA-1273)	SARS-CoV-2	Approved	Moderna	I.M.
mRNA-1010	Seasonal Influenza	Phase III Clinical Trial	Moderna	I.M.
mRNA-1345	Respiratory syncytial virus	Approved	Moderna	I.M.
mRNA-1893-P201	Zika virus	Phase II Clinical Trial Completed	Moderna	I.M.
mRNA-1647-P202	Cytomegalovirus infection	Phase II Clinical Trial Completed	Moderna	I.M.
CV-NCOV-004	SARS-CoV-2	Phase IIB/III Clinical Trial Completed	CureVac	I.M.
BNT162-01	SARS-CoV-2	Phase I, II Clinical Trial Completed	BioNTech–Pfizer	I.M.
mRNA-1273	SARS-CoV-2 B.1.351 variant	Phase II Clinical Trial Completed	Moderna	I.M.
mRNA-1083	Combine: influenza virus and SARS-CoV-2	Phase III Clinical TrialCompleted	Moderna	I.M.
IAVI G002(mRNA-1644; mRNA-1644v2-Core)	HIV-1	Phase I Clinical TrialActive, not recruiting	Moderna	I.M.
Cancer				
GO40558	Melanoma	Phase II Clinical Trial Completed	BioNTech– Genentech	I.V.
mRNA-4157-P201	Melanoma	Phase II Clinical Trial Recruiting	Moderna–Merck	I.M.
BNT111-01	Melanoma	Phase II Clinical Trial Active, not recruiting	BioNTech	I.V.
LUD2014-012-VAC	Non-small cell lung cancer	Phase II Clinical Trial Active, not recruiting	CureVac, Ludwig Institute	I.D.
GCT1046-01	Cancer	Phase I, II Clinical Trial Active, not recruiting	Genmab–BioNTech	I.V.
GCT1042-01	Solid tumors	Phase I, II Clinical Trial Active, not recruiting	Genmab–BioNTech	I.V.
BNT211-01	CLDN6+ tumors	Phase I Clinical Trial Active recruiting	BioNTech	I.V.
NCT06496373	Pancreatic Cancer Recurrent	Phase I Clinical Trial Active recruiting	Ruijin Hospital	I.V.
mRNA-4157	Unresectable solid tumors	Phase I Clinical Trial Active recruiting	Moderna	I.M.
RO7198457	Pancreatic Cancer	Phase I Clinical Trial Active not recruiting	BioNTech	I.V.
BNT112	Prostate Cancer	Phase I, II Clinical Trial, Terminated	BioNTech	I.V.
BNT113	Head and neck squamous cell carcinoma	Phase II, III Clinical Trial Active recruiting	BioNTech	I.V.
BNT152+153	Solid Tumor	Phase I Clinical Trial Active not recruiting	BioNTech	I.V.

Abbreviations: I.M., intramuscular; I.V., intravenous; I.D., intradermal.
